# Regular use of dental services among university students in southern Brazil

**DOI:** 10.11606/s1518-8787.2020054001935

**Published:** 2020-08-18

**Authors:** Mariana Silveira Echeverria, Alexandre Emidio Ribeiro Silva, Bernardo Antônio Agostini, Helena Silveira Schuch, Flávio Fernando Demarco

**Affiliations:** I Universidade Federal de Pelotas Faculdade de Medicina Programa de Pós-Graduação em Epidemiologia PelotasRS Brasil Universidade Federal de Pelotas. Faculdade de Medicina. Programa de Pós-Graduação em Epidemiologia. Pelotas, RS, Brasil; II Universidade Federal de Pelotas Faculdade de Odontologia Programa de Pós-Graduação em Odontologia PelotasRS Brasil Universidade Federal de Pelotas. Faculdade de Odontologia. Programa de Pós-Graduação em Odontologia. Pelotas, RS, Brasil; III IMED Faculdade de Odontologia Passo FundoRS Brasil IMED. Faculdade de Odontologia. Passo Fundo, RS, Brasil

**Keywords:** Young Adult, Dental Care, Oral Health, Health Services Accessibility, Socioeconomic Factors, Health Status Disparities

## Abstract

**OBJECTIVE:**

To verify the prevalence and factors associated with regular use of dental services in university students of the Universidade Federal de Pelotas (UFPel).

**METHODS:**

This cross-sectional study interviewed 1,865 students aged 18 years or older, starting bachelor’s degrees in 2017, enrolled in the second academic semester of 2017 and in the first of 2018 in classroom courses at UFPel. We considered regular users those who reported regularly going to the dentist with or without perceived dental problems. To test factors associated with regular use of dental services, demographic, socioeconomic and oral health variables were collected. Statistical analyses were based on Poisson regression models.

**RESULTS:**

The prevalence of regular use of dental services was 45.0% (95%CI 42.7–47.3). University students of high economic class (PR = 1.47; 95%CI 0.91–2.36), with last private dental appointment (PR = 1.29; 95%CI 1.03–1.61), positive self-perception of oral health (PR = 2.33; 95%CI 1.79–3.03) and no report of toothache in the last six months (PR = 1.22; 95%CI 1.03–1.45) showed higher prevalence of regular use of dental services.

**CONCLUSION:**

The results point to inequalities in the regular use of dental services related to socioeconomic factors and a lower use among university students with worse oral health conditions. These results suggest that public health prevention and promotion policies in higher education institutions must be carried out to ensure quality of life among these young adults.

## INTRODUCTION

In recent years, there has been an expansion of federal higher education institutions in Brazil. Federal universities are present in all units of the federation, with more than 1.1 million students enrolled^1^. Included in this scenario is the *Universidade Federal de Pelotas* (UFPel), which receives students from all Brazilian regions, with different demographic and socioeconomic backgrounds. These young adults are characterized as individuals undergoing transformation, entering adulthood and moving from their birth regions, and contextual factors can significantly influence their oral health^2^.

The social and racial quota policies introduced in Brazilian public universities in 2012 significantly increased the entry of young adults from more disadvantaged socioeconomic classes^3^. Considering this expansion, many of these individuals may have had difficulties in accessing health services, particularly oral health services, during their life trajectory.

Most studies addressing the use of dental services are based on Andersen’s^4^theoretical model, which aims to verify the association of contextual and individual social characteristics with different patterns of use of dental services^5^. The literature indicates that men, of black or brown skin color or indigenous people and individuals with lower education and socioeconomic class use oral health services less^6^. This fact can be explained, according to Hart^7^, by the “law of reverse care,” a phenomenon in which those who need health care most are the least likely to use it.

Besides the pattern of use of oral health services, the literature has underlined the importance of assessing the regularity of the habit of attending the dentist, identifying those who consult this professional when they present a problem or not and those who visit the dentist when still asymptomatic, for preventive reasons^8^. Regular use provides greater contact between patient and dentist, contributing to the knowledge, self-care and early detection of oral health problems^9^. There is already evidence of a positive association between regular use of dental services and better oral health condition^9^. In Brazil, the prevalence of regular use of these services ranged from 25.7% to 35.8% from 2009 to 2016 among adults^12,13^.

Considering that the majority of dental care expenses incurred by Brazilian families concern specialized procedures^14^ and that regular use of dental services results in less complex procedures^9^, we must encourage the use of dental services with adequate regularity and frequency, especially in a young population, to consolidate healthy behaviors. Thus, the present study aims to verify the prevalence and factors associated with the regular use of dental services in undergraduates entering UFPel in 2017.

## METHODS

This is a cross-sectional study that interviewed 1,865 students aged 18 years or older out of the 2,706 new students in 2017 enrolled in the second semester of 2017 and in the first semester of 2018 in classroom courses at UFPel. The university is located in the city of Pelotas (RS), in southern Brazil, and receives about 3,000 students annually, having 80 classroom courses with admission in the first semester of each year. Given this context, the present study is part of a census conducted at UFPel that sought information on the health and behaviors of university students.

This study is linked to the research consortium of the 2017 and 2018 Master’s students from the UFPel Graduate Program in Epidemiology. This strategy facilitates data collection, which occurs in a sole way with all master’s students conducting one common field work^15^. Data collection occurred between November 2017 and July 2018, by self-administered standardized questionnaires using RedCap software installed on tablets, answered in classrooms or other environments within the university.

The outcome “regular use of dental services” was measured by the question: “Which of the statements below describes your access to dental care? (0) I never go to the dentist. (1) I go to the dentist when I have a problem or when I know I need to have something fixed. (2) I go to the dentist occasionally, whether or not I have some kind of problem. (3) I go to the dentist regularly”^8^. Regular use was considered when the interviewee replied going to the dentist occasionally, with or without some kind of problem, or when they reported going to the dentist regularly.

To verify possible associated factors, we considered as demographic variables: gender (female and male) and self-declared skin color according to the Brazilian Institute of Geography and Statistics (white, black, brown or other). Regarding socioeconomic level, maternal education (illiterate, some elementary school, elementary school or some high school, high school or some college, college or some graduate studies and graduate studies) and economic class according to the Brazilian Economic Classification Criterion of the Brazilian Association of Research Companies – ABEP (A, B, C and D or E) were evaluated. To assess questions about students, we inquired on the region of residence prior to joining UFPel (South, Southeast or Midwest and North or Northeast), current housing situation (alone; with parents or other family members; with friends or colleagues; and with spouse, partner or significant other) and the area of knowledge of the course in which they were enrolled (exact and earth sciences, agrarian sciences and engineering; health and biological sciences; applied social and human sciences; linguistics, language and literature and arts). Finally, self-perceived oral health conditions – self-perceived oral health (excellent, very good or good and reasonable or poor) and presence of dental pain (yes or no) – and location of the last dental appointment (public or private) were considered to test factors associated with regular use of dental services.

The variables studied are based on Andersen’s classic theoretical model^4^. This model addresses the complexity of health services use in a comprehensive way, categorizing the determinants of service use into predisposing characteristics, enabling resources and factors related to the individual’s need. Predisposing factors, in turn, are divided into demographics, social structure and health beliefs. The present study included the participant’s gender as a demographic variable. Factors of social structure were considered: skin color, maternal education, economic class, region of origin and housing situation. The knowledge area variable aimed to reflect the differences in health beliefs, as it relates to the individuals’ values and knowledge about health and health services. As a enabling resource, related to the possibility of accessing the service, information on the location of the last dental appointment was used. Finally, to assess the need for treatment, which is the condition most proximal to services use, self-perceived oral health and dental pain were included.

**Figure 1:**
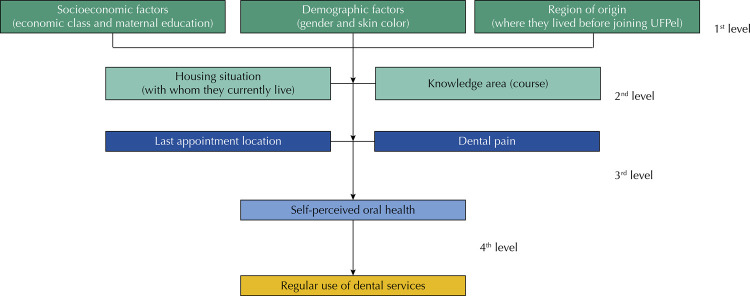
Analysis model. UFPel: Universidade Federal de Pelotas

Data were analyzed using the Stata^®^ 15.0 statistical package. Initially, descriptive analyses were performed using absolute and relative frequencies conducted according to the hierarchical analysis model described in Figure 1, by Poisson regression models. Adjustment modeling was performed by backward stepwise and all variables with p-value less than or equal to 0.2 were kept in the model. The significance level considered for all analyses was 5%. Missing data were estimated by the multiple imputation technique^16^.

The research project was submitted and approved by the Ethics and Research Committee of the *Faculdade de Medicina of the Universidade Federal de Pelotas* under protocol no. 79250317.0.0000.5317. All participants were previously informed about the study, signing an informed consent form, and the confidentiality of the information provided was guaranteed.

## RESULTS

Of the 2,706 eligible university students, 1,865 participated in the study, which corresponded to a response rate of 69%. Of these, the majority were female (54.8%), between 18 and 19 years old (41.5%) and white (72.1%). Most students reported their mother has high school (32.1%) and, according to ABEP, most students were classified as belonging to economic class B (44.2%). Half of the undergraduates live with their parents and/or siblings (50.4%) and many already lived in the country’s southern region prior to joining UFPel (83.3%). Most are currently enrolled in applied social and human sciences courses (34.4%). In 79.8% of the sample, university students perceived their oral health as excellent, very good or good; 71.9% had no toothache in the last six months, and most used private oral health services (82.8%). Descriptive analyses performed on the sample with multiple imputation showed similar percentages to those of the study sample, as presented in Tables 1 and 2.

**Table 1:** Description of the university students sample entering the *Universidade Federal de Pelotas* in 2017 according to demographic, socioeconomic and oral health characteristics. Pelotas, Rio Grande do Sul, Brazil, 2018.

Variables (n)	n	%
Gender (1,862)		
Female	1,021	54.83
Male	841	45.17
Age (1,852)		
18 to 19 years	768	41.47
20 to 22 years	603	32.56
23 years or older	481	25.97
Skin color (1,863)		
White	1,343	72.09
Black, brown or other	520	27.91
Mother’s schooling level (1,854)		
Illiterate	15	0.81
Elementary school	400	21.57
Elementary school/some high school	222	11.97
High school/some higher education	595	32.09
College/some graduate studies	410	22.11
Graduate studies	212	11.43
Region of origin (1,859)		
South	1,549	83.32
Southeast/Midwest	272	14.63
North/Northeast	38	2.04
Economic class (1,780)		
A	266	14.94
B	787	44.21
C	649	36.46
D/E	78	4.38
Knowledge area (1,865)		
Exact and earth sciences, agrarian sciences and engineering	544	29.17
Health and biological sciences	332	17.80
Applied social and human sciences	641	34.37
Linguistics, language and literature, and arts	348	18.66
Housing situation (1,861)		
Alone	234	12.57
With parents (father and/or mother and/or siblings)	937	50.35
With friends or colleagues	480	25.79
With spouse/partner/significant other	210	11.28
Regular use of dental services (1,854)		
No	1,020	55.02
Yes	834	44.98
Location of the last appointment (1,715)		
Public	295	17.20
Private	1,420	82.80
Self-perceived oral health (1,854)		
Excellent/very good/good	1,479	79.77
Moderate/poor	375	20.23
Toothache over the last six months (1,804)		
No	1,297	71.90
Yes	507	28.10

**Table 2:** Description of the imputed sample of university students enrolling at the *Universidade Federal de Pelotas* in 2017 according to demographic, socioeconomic and oral health characteristics. Pelotas, Rio Grande do Sul, Brazil, 2018.

Variables	%	95%CI
Gender		
Female	54.85	52.59–57.11
Male	45.15	42.88–47.41
Age		
18 to 19 years	41.45	39.20–43.70
20 to 22 years	32.56	30.42–34.70
23 years or older	25.99	23.99–27.99
Skin color		
White	72.07	70.00–74.10
Black, brown or other	27.93	25.89–29.97
Mother’s schooling level		
Illiterate	0.81	0.40–1.23
Some elementary school	21.58	19.70–23.45
Elementary school/some high school	11.98	10.50–13.46
High school/some college	32.10	29.97–34.23
College/some graduate studies	22.10	20.21–23.98
Graduate studies	11.42	9.97–12.87
Region of origin		
South	83.27	81.57–84.97
Southeast/Midwest	14.66	13.05–16.27
North/Northeast	2.06	1.41–2.71
Economic class		
A	14.81	13.17–16.45
B	43.95	41.63–46.26
C	36.71	34.45–38.97
D/E	4.53	3.57–5.50
Knowledge area		
Exact and earth sciences, agrarian sciences and engineering	29.17	27.10–31.23
Health and biological sciences	17.80	16.06–19.54
Applied social and human sciences	34.37	32.21–36.53
Linguistics, language and literature, and arts	18.66	16.89–20.43
Housing situation		
Alone	12.57	11.06–14.07
With parents (father and/or mother and/or siblings)	50.34	48.07–52.61
With friends or colleagues	25.77	23.78–27.76
With spouse/partner/significant other	11.32	9.88–12.76
Regular use of dental services		
No	55.05	52.78–57.32
Yes	44.95	42.68–47.21
Location of the last appointment		
Public	17.62	15.82–19.42
Private	82.38	80.58–84.18
Self-perceived oral health		
Excellent/very good/good	79.75	77.91–81.58
Moderate/poor	20.25	18.42–22.09
Toothache over the last six months		
No	71.75	69.66–73.83
Yes	28.25	26.17–30.34

95%CI: 95% confidence interval.

Regarding the regular use of dental services, the prevalence was 45.0% [95% confidence interval (95%CI) 42.7–47.3]. The demographic and socioeconomic variables positively associated with the regular use of dental services that showed statistical differences in the crude analysis were the high maternal education, living in the Southern region prior to joining UFPel, upper economic class and being enrolled in health or biological sciences courses. Regarding oral health variables, the absence of toothache, positive self-perceived oral health and use of private dental health services in the last appointment were associated with greater regular use of dental services. In the multiply imputed sample, besides the variables mentioned above, being white was associated with greater use of dental services on the crude analysis.

After adjustment for possible confounders, economic class, location of the last dental appointment, self-perceived oral health and toothache, remained associated with the outcome. The prevalence of regular use of dental services was 1.47 times higher among the wealthier than among the poorest. Among university students who consulted in private oral health services, the prevalence of regular use was 1.29 times higher than those who consulted with a dentist in public services. For self-perceived oral health, the prevalence of regular use of dental services was 2.33 times higher in those who reported good oral health compared with those who perceived their own oral health as negative. University students who did not report dental pain had a 1.22 times higher prevalence of regular use of dental services than students who had dental pain in the last six months. Table 3 presents the crude and adjusted analysis of the factors associated with the regular use of dental services in the actual study sample. In the adjusted analysis using the imputed data sample, all variables that showed statistically significant association in the study sample remained after imputation. Table 4 shows the association analyses performed on the imputed sample.

**Table 3:** Prevalence ratios (PR) and 95% confidence intervals (95%CI) of the crude and adjusted analysis of regular use of dental services according to demographic, socioeconomic and oral health variables of the study participants. Pelotas, Rio Grande do Sul, Brazil, 2018.

Variables	Regular use of dental services			
	Crude analysis		Adjusted analysis*	
	PR (95%CI)	p	PR (95%CI)	p
Gender				
Male	1.00	0.780		
Female	1.02 (0.89–1.170)			
Skin color				
Black, brown or other	1.00	0.055		
White	1.17 (1.00–1.33)			
Mother’s schooling level				
Illiterate	1.00	0.002		
Some elementary school	1.43 (0.53–3.85)			
Elementary school/some high school	1.53 (0.56–4.15)			
High school/some college	1.42 (0.53–3.81)			
College/some graduate studies	1.81 (0.67–4.87)			
Graduate studies	1.95 (0.72–5.28)			
Region of origin				
North/Northeast	1.00	0.006	1.00	0.085
South	1.98 (1.02–3.81)		1.50 (0.78–2.90)	
Southeast/Midwest	1.60 (0.81–3.17)		1.32 (0.67–2.62)	
Economic class				
D/E	1.00	< 0.001	1.00	0.025
A	2.12 (1.36–3.31)		1.47 (0.91–2.36)	
B	1.66 (1.08–2.55)		1.27 (0.80–2.00)	
C	1.41 (0.91–2.18)		1.19 (0.75–1.88)	
Knowledge area				
Linguistics, language and literature, and arts	1.00	0.003	1.00	
Exact and earth sciences, agrarian sciences and engineering	1.33 (1.08–1.65)		1.24 (0.99–1.55)	0.080
Health and biological sciences	1.50 (1.20–1.89)		1.25 (0.98–1.60)	
Applied social and human sciences	1.18 (0.96–1.47)		1.17 (0.93–1.46)	
Housing situation				
With friends or colleagues	1.00	0.125		
Alone	1.19 (0.94–1.50)			
With parents (father and/or mother and/or siblings)	1.16 (0.98–1.38)			
With spouse/partner/significant other	1.06 (0.83–1.36)			
Location of the last appointment				
Public	1.00	< 0.001	1.00	0.027
Private	1.46 (1.19–1.81)		1.29 (1.03–1.61)	
Self-perceived oral health				
Negative	1.00	< 0.001	1.00	< 0.001
Positive	2.57 (2.02–3.25)		2.33 (1.79–3.03)	
Toothache				
Yes	1.00	< 0.001	1.00	0.023
No	1.38 (1.17–1.63)		1.22 (1.03–1.45)	

* Analysis adjusted for gender, skin color, economic class, maternal education, region of origin, area of knowledge, current housing situation, location of last dental appointment, dental pain and self-perceived oral health.

**Table 4:** Prevalence ratios (PR) and 95% confidence intervals (95%CI) of the crude and adjusted analysis of regular use of dental services according to demographic, socioeconomic and oral health variables of the study participants (imputed sample). Pelotas, Rio Grande do Sul, Brazil, 2018.

Variables	Regular use of dental services			
	Crude analysis		Adjusted analysis*	
	PR (95%CI)	p	PR (95%CI)	p
Gender				
Male	1.00	0.691		
Female	1.02 (0.92–1.13)			
Skin color				
Black, brown or other	1.00	0.014		
White	1.16 (1.03–1.31)			
Mother’s schooling level				
Illiterate	1.00	< 0.001		
Some elementary school	1.45 (0.62–3.38)			
Elementary school/some high school	1.56 (0.66–3.64)			
High school/some college	1.44 (0.62–3.35)			
College/some graduate studies	1.84 (0.79–4.27)			
Graduate studies	1.98 (0.85–4.62)			
Region of origin				
North/Northeast	1.00	0.003	1.00	0.021
South	2.00 (1.12–3.55)		1.80 (0.93–3.49)	
Southeast/Midwest	1.62 (0.90–2.93)		1.54 (0.78–3.06)	
Economic class				
D/E	1.00	< 0.001	1.00	0.003
A	2.08 (1.44–3.00)		1.65 (1.06–2.58)	
B	1.63 (1.14–2.33)		1.38 (0.90–2.12)	
C	1.39 (0.97–2.00)		1.28 (0.83–1.96)	
Knowledge area				
Linguistics, language and literature, and arts	1.00	< 0.001	1.00	
Exact and earth sciences, agrarian sciences and engineering	1.34 (1.13–1.58)		1.22 (0.99–1.52)	0.062
Health and biological sciences	1.51 (1.27–1.79)		1.30 (1.03–1.63)	
Applied social and human sciences	1.19 (1.01–1.41)		1.15 (0.93–1.42)	
Housing situation				
With friends or colleagues	1.00	0.092		
Alone	1.18 (1.00–1.41)			
With parents (father and/or mother and/or siblings)	1.16 (1.02–1.32)			
With spouse/partner/significant other	1.05 (0.87–1.28)			
Location of the last appointment				
Public	1.00	< 0.001	1.00	0.016
Private	1.50 (1.26–1.78)		1.30 (1.05–1.61)	
Self-perceived oral health				
Negative	1.00	< 0.001	1.00	< 0.001
Positive	2.57 (2.09–3.17)		2.28 (1.79–2.90)	
Toothache				
Yes	1.00	< 0.001	1.00	0.028
No	1.38 (1.20–1.57)		1.20 (1.02–1.43)	

* Analysis adjusted for gender, skin color, economic class, maternal education, region of origin, area of knowledge, current housing situation, location of last dental appointment, dental pain and self-perceived oral health.

## DISCUSSION

The 45.0% prevalence of regular use of dental services found in this university-based study was higher than that reported in other population-based surveys conducted in Brazil in the same age group. The authors are unaware of other studies that have addressed the issue of regular use of dental services specifically in the Brazilian university population. In Pelotas, city where UFPel is located, a population-based study conducted by Camargo et al.^17^ in 2009 used the same question to measure regular use of dental services and found a prevalence of 32.8% in adults. In a more recent study, also population-based, conducted in Minas Gerais, the prevalence was 35.8% for regular use of dental services among individuals over 18 years of age^13^. In this study, students who had their last dental appointment more than a year ago were classified as irregular users of dental services. Among those who had their last dental appointment less than a year ago, the regular use of dental services was defined based on two questions: one on the frequency/regularity of appointments and the other on the reason for the consultation. Students who visited the dentist in the last year and reported regular use in both subsequent questions were classified as regularly using dental services.

It is important to note that previous studies used population-based samples, while our study sample comprises university students. Despite the university inclusion policies adopted in recent years, our study does not reflect the Brazilian population profile in this age group, as it excludes populations in greater social vulnerability, which have the greatest oral health problems and less access to health services^18^. However, in Brazil, there was an expansion of policies for higher education in the last two decades, with the inclusion of groups historically excluded from this educational level, which significantly changed the profile of undergraduate students. Although the democratization process of Brazilian higher education can be observed, its access system remains elitist^3^. The higher prevalence found in the present study may reflect this inclusion and change in the profile of Brazilian university students, highlighting a policy favoring social equity.

The association between economic class and regular use of dental services observed in this study agrees with the current literature, according to which poorer individuals show less use and greater difficulties in accessing dental services^11^. In a study conducted with 18 selected countries from the Organization for Economic Cooperation and Development (OECD), all of them presented significant inequalities in access to dental services related to income^19^. Thus, economic class is an important predictor in accessing information about preventive health habits and behaviors^20^. Additionally, higher income results in the possibility of purchasing health services^14^. When analyzing the global data on health expenditures, the countries with the highest income *per capita* are those with the highest expenditures on oral health^21^. The same process that occurs between countries occurs between individuals^22^. As such, regular use of dental services among the most economically vulnerable individuals becomes even more difficult.

Demographic factors such as gender and skin color were associated with regular use of dental services in several parts of the world, according to the systematic review and meta-analysis conducted by Reda et al^11^. However, our study found a statistical association with skin color in the crude analysis only when dealing with losses by multiple imputation of missing data, which shows that this association possibly exists, but we were unable to find it. For gender, not even the imputation analysis yielded in a positive statistical association, which suggests that particularities in the university population’s characteristics may justify the absence of association between gender and regular use of dental services.

Regarding self-reported oral health conditions, a positive perception and absence of dental pain were associated with regular use of dental services in this study. Corroborating these findings, the literature shows that positive self-perceived oral health^12,13,17^ and the absence of dental pain^13^ were associated with greater regular use of dental services. These results can be explained by the fact that health perception may act as a determinant for understanding the importance of regular dentist visits and, consequently, more preventive actions result in fewer episodes of pain and a positive self-perceived oral health.

The greater regular use of oral services was associated with the location of the last dental appointment. In Brazil, Machado^12^ found a higher prevalence of regular use in those who used private oral health services than in users of public institutions. After a reform in public oral health services, Finland saw an increase in the use of these services in the last 12 months, besides an increase in public care in relation to the private market^23^.

The differential losses and refusals, higher among older males enrolled in courses in the area of exact and earth sciences, agrarian sciences and engineering and who lived in the southern region prior to joining UFPel, which could result in selection bias, are limitations of the present study. Due to its cross-sectional design, reverse causality bias may occur for the associations between regular use of dental services and oral health variables, which can be two-way. Another possible limitation concerns obtaining self-reported information on the regularity of dentist appointments. Considering that this is a socially desirable behavior, there is the possibility of overestimating the report of dentist appointments for preventive reasons.

The way regular use of oral health services was assessed is a strength of this study, since most studies addressing the issue only investigate the recall time of the last appointment to characterize regularly visiting the dentist, without distinguishing between the individual’s motivation. In addition, the originality of the studied population stands out as a strong point.

Finally, in this study, students reported using dental services more to solve oral health problems and not regularly to prevent them, as would be desirable. The results point to inequalities in the regular use of dental services related to socioeconomic factors and a lower use among university students with worse oral health conditions. These results suggest that public health prevention and promotion policies in higher education institutions must be carried out to ensure quality of life for this population.
