# Supplementary material for: Regular use of dental services among university students in southern Brazil
Source: Rev Saude Publica. 2020 Aug 18;54:85. doi: 10.11606/s1518-8787.2020054001935 (PMC7438030; doi:10.11606/s1518-8787.2020054001935)
Supplement: Translation

# Translation: Uso regular de serviços odontológicos entre alunos de uma universidade do sul do Brasil

**Authors:** Mariana Silveira Echeverria, Alexandre Emidio Ribeiro Silva, Bernardo Antônio Agostini, Helena Silveira Schuch, Flávio Fernando Demarco

**Affiliations:** I Brasil Universidade Federal de Pelotas. Faculdade de Medicina. Programa de Pós-Graduação em Epidemiologia. Pelotas, RS, Brasil; II Brasil Universidade Federal de Pelotas. Faculdade de Odontologia. Programa de Pós-Graduação em Odontologia. Pelotas, RS, Brasil; III Brasil IMED. Faculdade de Odontologia. Passo Fundo, RS, Brasil

**Keywords:** Adulto Jovem, Assistência Odontológica, Saúde Bucal, Acesso aos Serviços de Saúde, Fatores Socioeconômicos, Disparidades nos Níveis de Saúde

## Abstract

**OBJETIVO:**

Verificar a prevalência e os fatores associados ao uso regular de serviços odontológicos em acadêmicos da Universidade Federal de Pelotas (UFPel).

**MÉTODOS:**

Este estudo transversal entrevistou 1.865 estudantes de 18 anos ou mais de idade, ingressantes em 2017, matriculados no segundo semestre letivo de 2017 e no primeiro de 2018 em cursos presenciais da UFPel. Consideraram-se usuários regulares os indivíduos que relataram ir regularmente ao dentista com ou sem problemas dentários percebidos. A fim de testar fatores associados ao uso regular de serviços odontológicos, foram coletadas variáveis demográficas, socioeconômicas e de saúde bucal. As análises estatísticas foram baseadas em modelos de regressão de Poisson.

**RESULTADOS:**

A prevalência de uso regular de serviços odontológicos foi de 45,0% (IC95% 42,7–47,3). Os universitários de classe econômica elevada (RP = 1,47; IC95% 0,91–2,36), com última consulta odontológica particular (RP = 1,29; IC95% 1,03–1,61), autopercepção positiva da saúde bucal (RP = 2,33; IC95% 1,79–3,03) e sem relato de dor de dente nos últimos seis meses (RP = 1,22; IC95% 1,03–1,45) apresentaram maiores prevalências de uso regular dos serviços odontológicos.

**CONCLUSÃO:**

Os resultados apontam para desigualdades no uso regular dos serviços odontológicos relacionadas a fatores socioeconômicos e um menor uso entre os universitários com piores condições de saúde bucal. Esses resultados sugerem que políticas públicas de prevenção e promoção em saúde em instituições de ensino superior devem ser realizadas para garantir qualidade de vida entre esses jovens.

## INTRODUÇÃO

Nos últimos anos houve uma ampliação das instituições federais de ensino superior no Brasil. As universidades federais estão presentes em todas as unidades da federação, com mais de 1,1 milhão de alunos matriculados^1^. Nesse cenário está a Universidade Federal de Pelotas (UFPel), a qual recebe discentes de todas as regiões brasileiras, com diferentes características demográficas e socioeconômicas. Esses jovens caracterizam-se como indivíduos em fase de transformação, entrando na vida adulta e deslocando-se de suas regiões de nascimento, sendo que fatores contextuais podem influenciar significativamente a sua saúde bucal^2^.

Além disso, as políticas de cotas sociais e raciais introduzidas nas universidades públicas brasileiras em 2012 aumentaram significativamente o ingresso de jovens de classes socioeconômicas mais desfavorecidas^3^. Considerando essa expansão, muitos desses indivíduos podem ter tido dificuldades no acesso a serviços de saúde, particularmente os de saúde bucal, durante a sua trajetória de vida.

A maioria dos estudos que abordam a utilização de serviços odontológicos é baseada no modelo teórico proposto por Andersen^4^. O modelo dispõe-se a verificar a associação de características sociais contextuais e individuais com diferentes padrões de utilização de serviços odontológicos^5^. A literatura indica que homens, negros, pardos ou indígenas e indivíduos com escolaridade e classe socioeconômica mais baixas utilizam menos os serviços de saúde bucal^6^. Esse fato pode ser explicado, segundo Hart^7^, pela “lei dos cuidados inversos”, fenômeno em que aqueles que mais necessitam de cuidados em saúde são os menos propensos a utilizá-los.

Além do padrão de utilização dos serviços de saúde bucal, a literatura tem apontado para a importância de avaliar a regularidade do hábito de frequentar o dentista, identificando aqueles indivíduos que consultam esse profissional quando apresentam ou não um problema e aqueles que procuram o dentista quando ainda assintomáticos, com a finalidade de realizar uma revisão^8^. O uso regular proporciona um maior contato entre o paciente e o dentista, contribuindo para o conhecimento, autocuidado e detecção precoce de problemas relacionados à saúde bucal^9^. Já há evidências de uma associação positiva entre o uso regular de serviços odontológicos e melhor condição de saúde bucal^9^. No Brasil, a prevalência de uso regular desses serviços variou de 25,7% a 35,8% de 2009 a 2016 entre adultos^12,13^.

Considerando que a maioria dos gastos com assistência odontológica realizados pelas famílias brasileiras são referentes a procedimentos especializados^14^ e que o uso regular de serviços odontológicos resulta em procedimentos menos complexos^9^, é necessário estimular a utilização dos serviços odontológicos com periodicidade e frequência adequadas, especialmente em uma população jovem, para consolidar comportamentos saudáveis. Por essa razão, o presente estudo tem como objetivo verificar a prevalência e os fatores associados ao uso regular de serviços odontológicos em uma população universitária ingressante em 2017 na UFPel.

## MÉTODOS

Trata-se de um estudo transversal que entrevistou 1.865 estudantes de 18 anos ou mais de idade dos 2.706 ingressantes em 2017 matriculados no segundo semestre letivo de 2017 e no primeiro semestre de 2018 em cursos presenciais da UFPel. A universidade está localizada na cidade de Pelotas (RS), no sul do Brasil, e recebe anualmente cerca de 3.000 ingressantes, possuindo 80 cursos presenciais com ingresso no primeiro semestre letivo de cada ano. Nesse contexto, o presente trabalho faz parte de um censo realizado na UFPel que buscou informações sobre a saúde e comportamentos de estudantes universitários.

Este estudo está vinculado ao consórcio de pesquisa da turma de mestrandos de 2017 e 2018 do Programa de Pós-Graduação em Epidemiologia da UFPel. Essa estratégia facilita a coleta dos dados, que ocorre de forma única com todos os mestrandos realizando um trabalho de campo comum^15^. A coleta de dados ocorreu entre novembro de 2017 e julho de 2018, por meio de questionários padronizados autoaplicados utilizando o software RedCap instalado em tablets, respondidos nas salas de aula ou em outros ambientes dentro da universidade.

O desfecho “uso regular de serviços odontológicos” foi medido por meio da pergunta: “Qual das afirmações abaixo descreve o seu acesso aos cuidados odontológicos? (0) Eu nunca vou ao dentista. (1) Eu vou ao dentista quando eu tenho um problema ou quando sei que preciso ter alguma coisa arrumada. (2) Eu vou ao dentista ocasionalmente, tenha ou não algum tipo de problema. (3) Eu vou ao dentista regularmente”^8^. O uso regular foi considerado quando o entrevistado respondeu que vai ao dentista ocasionalmente, tenha ou não algum tipo de problema, ou quando relatou ir ao dentista regularmente.

Para verificar possíveis fatores associados, foram consideradas as seguintes variáveis demográficas: sexo (feminino e masculino) e cor da pele autodeclarada de acordo com Instituto Brasileiro de Geografia e Estatística (branca e preta, parda ou outra). Em relação ao nível socioeconômico, foram avaliadas a escolaridade materna (analfabeta, ensino fundamental incompleto, ensino fundamental completo ou médio incompleto, ensino médio completo ou superior incompleto, ensino superior completo ou pós-graduação incompleta e pós-graduação completa) e a classe econômica segundo o Critério de Classificação Econômica Brasil da Associação Brasileira de Empresas de Pesquisa – ABEP (A, B, C e D ou E). A fim de avaliar questões sobre os estudantes, foi perguntada a região de moradia no período anterior ao ingresso na UFPel (Sul, Sudeste ou Centro-Oeste e Norte ou Nordeste), situação atual de moradia (sozinho; com os pais ou outros familiares; com amigos ou colegas; e com cônjuge, companheiro ou namorado) e a área do conhecimento do curso no qual está matriculado (ciências exatas e da terra, ciências agrárias e engenharias; ciências da saúde e biológicas; ciências sociais aplicadas e humanas; e linguística, letras e artes). Por fim, as condições autopercebidas de saúde bucal – autopercepção da saúde bucal (excelente, muito boa ou boa e razoável ou ruim) e presença de dor de origem dentária (não ou sim) – e o local da última consulta odontológica (público ou privado) foram considerados para testar os fatores associados ao uso regular dos serviços odontológicos.

As variáveis estudadas estão embasadas no modelo teórico clássico de Andersen^4^. Esse modelo aborda a complexidade no uso de serviços de saúde de maneira bastante abrangente, categorizando os determinantes do uso de serviços em características predisponentes, recursos capacitantes e fatores relacionados à necessidade do indivíduo. Os fatores predisponentes, por sua vez, se dividem em demográficos, de estrutura social e crenças em saúde. Neste estudo, como variável demográfica foi incluído o sexo do participante. Foram considerados fatores de estrutura social a cor da pele, a escolaridade materna, a classe econômica, a região do país de origem e o padrão de moradia. Já a variável de área de conhecimento visou refletir as diferenças crenças em saúde, já que se relaciona a valores e conhecimentos dos indivíduos sobre saúde e serviços de saúde. Como recurso capacitante, relacionado à possibilidade de acesso ao serviço, foi utilizada a informação sobre o local da última consulta odontológica. Por fim, para avaliar a necessidade de tratamento, que é a condição mais proximal ao uso de serviços, foram incluídas a autopercepção de saúde bucal e a dor dentária.

Os dados foram analisados com o auxílio do pacote estatístico Stata^®^ 15.0. Inicialmente, foram realizadas as análises descritivas por meio de frequências absolutas e relativas. As análises foram executadas conforme o modelo de análise hierarquizado descrito na Figura, por regressão de Poisson. A modelagem de ajuste se deu pela técnica *backward* s*tepwise* e foram mantidas no modelo todas as variáveis com p-valor menor ou igual a 0,2. Para todas as análises, foi considerado um nível de significância de 5%. Os dados faltantes foram estimados pela técnica de imputação múltipla^16^.

**Figura:** Modelo de análise. UFPel: Universidade Federal de Pelotas

O projeto de pesquisa foi submetido e aprovado no Comitê de Ética e Pesquisa da Faculdade de Medicina da Universidade Federal de Pelotas sob o protocolo número 79250317.0.0000.5317. Todos os participantes foram esclarecidos previamente sobre o estudo e foi garantido a eles o sigilo das informações prestadas. Além disso, foi obtido de cada participante um termo de consentimento livre e esclarecido.

## RESULTADOS

Dos 2.706 universitários elegíveis, participaram do estudo 1.865, o que correspondeu a uma taxa de resposta de 69%. Desses, a maioria era do sexo feminino (54,8%), tinha entre 18 e 19 anos de idade (41,5%) e cor da pele branca (72,1%). A maioria dos estudantes relatou que a mãe possui ensino médio completo (32,1%) e, de acordo com a ABEP, a maior parte dos discentes foi classificada como pertencente à classe econômica B (44,2%). Metade dos universitários moram com os pais e/ou irmãos (50,4%) e muitos já viviam na região Sul do país no período anterior ao ingresso na UFPel (83,3%). A maior parte encontra-se atualmente matriculada em cursos das ciências sociais aplicadas e humanas (34,4%). Em 79,8% da amostra, os universitários autoperceberam sua saúde bucal como excelente, muito boa ou boa. Além disso, 71,9% não tiveram dor de dente nos últimos seis meses, e a maioria utilizou serviços privados de saúde bucal (82,8%). Ademais, as análises descritivas realizadas na amostra com imputação múltipla mostraram percentuais similares aos da amostra do estudo, o que pode ser observado nas Tabelas 1 e 2.

**Tabela 1:** Descrição da amostra de universitários ingressantes em 2017 na Universidade Federal de Pelotas segundo características demográficas, socioeconômicas e de saúde bucal. Pelotas, Rio Grande do Sul, Brasil, 2018.

Variáveis (n)	n	%
Sexo (1.862)		
Feminino	1.021	54,83
Masculino	841	45,17
Idade (1.852)		
18 a 19 anos	768	41,47
20 a 22 anos	603	32,56
23 anos ou mais	481	25,97
Cor da pele (1.863)		
Branca	1.343	72,09
Preta, parda ou outra	520	27,91
Escolaridade materna (1.854)		
Analfabeta	15	0,81
Ensino fundamental incompleto	400	21,57
Ensino fundamental completo/médio incompleto	222	11,97
Ensino médio completo/superior incompleto	595	32,09
Ensino superior completo/pós-graduação incompleta	410	22,11
Pós-graduação completa	212	11,43
Região de origem (1.859)		
Sul	1.549	83,32
Sudeste/Centro-Oeste	272	14,63
Norte/Nordeste	38	2,04
Classe econômica (1.780)		
A	266	14,94
B	787	44,21
C	649	36,46
D/E	78	4,38
Área do conhecimento (1.865)		
Ciências exatas e da terra, ciências agrárias e engenharias	544	29,17
Ciências da saúde e biológicas	332	17,80
Ciências sociais aplicadas e humanas	641	34,37
Linguística, letras e artes	348	18,66
Situação de moradia (1.861)		
Sozinho(a)	234	12,57
Com os pais (pai e/ou mãe e/ou irmãos)	937	50,35
Com amigos(as) ou colegas	480	25,79
Com cônjuge/companheiro(a)/namorado(a)	210	11,28
Uso regular dos serviços odontológicos (1.854)		
Não	1.020	55,02
Sim	834	44,98
Local da última consulta odontológica (1.715)		
Público	295	17,20
Privado	1.420	82,80
Autopercepção da saúde bucal (1.854)		
Excelente/muito boa/boa	1.479	79,77
Razoável/ruim	375	20,23
Dor de dente nos últimos seis meses (1.804)		
Não	1.297	71,90
Sim	507	28,10

**Tabela 2:** Descrição da amostra imputada de universitários ingressantes em 2017 na Universidade Federal de Pelotas segundo características demográficas, socioeconômicas e de saúde bucal. Pelotas, Rio Grande do Sul, Brasil, 2018.

Variáveis	%	IC95%
Sexo		
Feminino	54,85	52,59–57,11
Masculino	45,15	42,88–47,41
Idade		
18 a 19 anos	41,45	39,20–43,70
20 a 22 anos	32,56	30,42–34,70
23 anos ou mais	25,99	23,99–27,99
Cor da pele		
Branca	72,07	70,00–74,10
Preta, parda ou outra	27,93	25,89–29,97
Escolaridade materna		
Analfabeta	0,81	0,40–1,23
Ensino fundamental incompleto	21,58	19,70–23,45
Ensino fundamental completo/médio incompleto	11,98	10,50–13,46
Ensino médio completo/superior incompleto	32,10	29,97–34,23
Ensino superior completo/pós-graduação incompleta	22,10	20,21–23,98
Pós-graduação completa	11,42	9,97–12,87
Região de origem		
Sul	83,27	81,57–84,97
Sudeste/Centro-Oeste	14,66	13,05–16,27
Norte/Nordeste	2,06	1,41–2,71
Classe econômica		
A	14,81	13,17–16,45
B	43,95	41,63–46,26
C	36,71	34,45–38,97
D/E	4,53	3,57–5,50
Área do conhecimento		
Ciências exatas e da terra, ciências agrárias e engenharias	29,17	27,10–31,23
Ciências da saúde e biológicas	17,80	16,06–19,54
Ciências sociais aplicadas e humanas	34,37	32,21–36,53
Linguística, letras e artes	18,66	16,89–20,43
Situação de moradia		
Sozinho(a)	12,57	11,06–14,07
Com os pais (pai e/ou mãe e/ou irmãos)	50,34	48,07–52,61
Com amigos(as) ou colegas	25,77	23,78–27,76
Com cônjuge/companheiro(a)/namorado(a)	11,32	9,88–12,76
Uso regular dos serviços odontológicos		
Não	55,05	52,78–57,32
Sim	44,95	42,68–47,21
Local da última consulta odontológica		
Público	17,62	15,82–19,42
Privado	82,38	80,58–84,18
Autopercepção da saúde bucal		
Excelente/muito boa/boa	79,75	77,91–81,58
Razoável/ruim	20,25	18,42–22,09
Dor de dente nos últimos seis meses		
Não	71,75	69,66–73,83
Sim	28,25	26,17–30,34

IC95%: intervalo de confiança de 95%

Quanto ao uso regular dos serviços odontológicos, a prevalência foi de 45,0% (intervalo de confiança de 95% [IC95%] 42,7–47,3). As variáveis demográficas e socioeconômicas associadas positivamente ao uso regular de serviços odontológicos que apresentaram diferenças estatísticas na análise bruta foram a elevada escolaridade materna, viver na região Sul do país no período anterior ao ingresso na UFPel, a classe econômica alta e estar matriculado em cursos da área da saúde ou das ciências biológicas. Em relação às variáveis de saúde bucal, a ausência de dor de dente, autopercepção de saúde bucal positiva e utilização de serviços de saúde odontológicos privados na última consulta foram associadas ao maior uso regular de serviços odontológicos. Na amostra imputada, além das variáveis acima mencionadas, a cor da pele branca esteve associada ao maior uso de serviços odontológicos quando da análise bruta.

Após análise ajustada para os possíveis confundidores, permaneceram associadas ao desfecho a classe econômica, local da última consulta odontológica, autopercepção da saúde bucal e dor de dente. A prevalência do uso regular de serviços odontológicos foi 1,47 vez maior entre os mais ricos que entre os mais pobres. Entre os universitários que se consultaram em serviços particulares de saúde bucal, a prevalência de uso regular foi 1,29 vez maior que os que se consultaram com um dentista em serviços públicos. Para autopercepção de saúde bucal, a prevalência de uso regular de serviços odontológicos foi 2,33 vezes maior em quem relatou uma boa saúde bucal comparado aos que percebem a própria saúde bucal como negativa. Além disso, os universitários que não relataram dor dentária apresentaram uma prevalência 1,22 vez maior de uso regular dos serviços odontológicos que os estudantes que tiveram dor dentária nos últimos seis meses. A análise bruta e ajustada dos fatores associados ao uso regular dos serviços odontológicos na amostra real do estudo pode ser observada na Tabela 3. Na análise ajustada utilizando a amostra de dados imputados, todas as variáveis que na amostra do estudo tiveram associação estatisticamente significativas permaneceram após a imputação. As análises de associação realizadas na amostra imputada podem ser observadas na Tabela 4.

**Tabela 3:** Razões de prevalência (RP) e intervalos de confiança de 95% (IC95%) da análise bruta e ajustada do uso regular dos serviços odontológicos segundo variáveis demográficas, socioeconômicas e de saúde bucal dos participantes do estudo. Pelotas, Rio Grande do Sul, Brasil, 2018.

Variáveis	Uso regular dos serviços odontológicos			
	Análise bruta		Análise ajustada*	
	RP (IC95%)	p	RP (IC95%)	p
Sexo				
Masculino	1,00	0,780		
Feminino	1,02 (0,89–1,17)			
Cor da pele				
Preta, parda ou outra	1,00	0,055		
Branca	1,17 (1,00–1,33)			
Escolaridade materna				
Analfabeta	1,00	0,002		
Ensino fundamental incompleto	1,43 (0,53–3,85)			
Ensino fundamental completo/médio incompleto	1,53 (0,56–4,15)			
Ensino médio completo/superior incompleto	1,42 (0,53–3,81)			
Ensino superior completo/pós-graduação incompleta	1,81 (0,67–4,87)			
Pós-graduação completa	1,95 (0,72–5,28)			
Região de origem				
Norte/Nordeste	1,00	0,006	1,00	0,085
Sul	1,98 (1,02–3,81)		1,50 (0,78–2,90)	
Sudeste/Centro-Oeste	1,60 (0,81–3,17)		1,32 (0,67–2,62)	
Classe econômica				
D/E	1,00	< 0,001	1,00	0,025
A	2,12 (1,36–3,31)		1,47 (0,91–2,36)	
B	1,66 (1,08–2,55)		1,27 (0,80–2,00)	
C	1,41 (0,91–2,18)		1,19 (0,75–1,88)	
Área do conhecimento				
Linguística, letras e artes	1,00	0,003	1,00	
Ciências exatas e da terra, ciências agrárias e engenharias	1,33 (1,08–1,65)		1,24 (0,99–1,55)	0,080
Ciências da saúde e biológicas	1,50 (1,20–1,89)		1,25 (0,98–1,60)	
Ciências sociais aplicadas e humanas	1,18 (0,96–1,47)		1,17 (0,93–1,46)	
Situação de moradia				
Com amigos(as) ou colegas	1,00	0,125		
Sozinho(a)	1,19 (0,94–1,50)			
Com os pais (pai e/ou mãe e/ou irmãos)	1,16 (0,98–1,38)			
Com cônjuge/companheiro(a)/namorado(a)	1,06 (0,83–1,36)			
Local da última consulta odontológica				
Público	1,00	< 0,001	1,00	0,027
Privado	1,46 (1,19–1,81)		1,29 (1,03–1,61)	
Autopercepção da saúde bucal				
Negativa	1,00	< 0,001	1,00	< 0,001
Positiva	2,57 (2,02–3,25)		2,33 (1,79–3,03)	
Dor de dente				
Sim	1,00	< 0,001	1,00	0,023
Não	1,38 (1,17–1,63)		1,22 (1,03–1,45)	

* Análise ajustada para sexo, cor da pele, classe econômica, escolaridade materna, região de origem, área do conhecimento, situação de moradia atual, local da última consulta odontológica, dor dentária e autopercepção da saúde bucal.

**Tabela 4:** Razões de prevalência (RP) e intervalos de 95% de confiança (IC95%) da análise bruta e ajustada do uso regular dos serviços odontológicos segundo variáveis demográficas, socioeconômicas e de saúde bucal dos participantes do estudo (amostra imputada). Pelotas, Rio Grande do Sul, Brasil, 2018.

Variáveis	Uso regular dos serviços odontológicos			
	Análise bruta		Análise ajustada*	
	RP (IC95%)	p	RP (IC95%)	p
Sexo				
Masculino	1,00	0,691		
Feminino	1,02 (0,92–1,13)			
Cor da pele				
Preta, parda ou outra	1,00	0,014		
Branca	1,16 (1,03–1,31)			
Escolaridade materna				
Analfabeta	1,00	< 0,001		
Ensino fundamental incompleto	1,45 (0,62–3,38)			
Ensino fundamental completo/médio incompleto	1,56 (0,66–3,64)			
Ensino médio completo/superior incompleto	1,44 (0,62–3,35)			
Ensino superior completo/pós-graduação incompleta	1,84 (0,79–4,27)			
Pós-graduação completa	1,98 (0,85–4,62)			
Região de origem				
Norte/Nordeste	1,00	0,003	1,00	0,021
Sul	2,00 (1,12–3,55)		1,80 (0,93–3,49)	
Sudeste/Centro-Oeste	1,62 (0,90–2,93)		1,54 (0,78–3,06)	
Classe econômica				
D/E	1,00	< 0,001	1,00	0,003
A	2,08 (1,44–3,00)		1,65 (1,06–2,58)	
B	1,63 (1,14–2,33)		1,38 (0,90–2,12)	
C	1,39 (0,97–2,00)		1,28 (0,83–1,96)	
Área do conhecimento				
Linguística, letras e artes	1,00	< 0,001	1,00	
Ciências exatas e da terra, ciências agrárias e engenharias	1,34 (1,13–1,58)		1,22 (0,99–1,52)	0,062
Ciências da saúde e biológicas	1,51 (1,27–1,79)		1,30 (1,03–1,63)	
Ciências sociais aplicadas e humanas	1,19 (1,01–1,41)		1,15 (0,93–1,42)	
Situação de moradia				
Com amigos(as) ou colegas	1,00	0,092		
Sozinho(a)	1,18 (1,00–1,41)			
Com os pais (pai e/ou mãe e/ou irmãos)	1,16 (1,02–1,32)			
Com cônjuge/companheiro(a)/namorado(a)	1,05 (0,87–1,28)			
Local da última consulta odontológica				
Público	1,00	< 0,001	1,00	0,016
Privado	1,50 (1,26–1,78)		1,30 (1,05–1,61)	
Autopercepção da saúde bucal				
Negativa	1,00	< 0,001	1,00	< 0,001
Positiva	2,57 (2,09–3,17)		2,28 (1,79–2,90)	
Dor de dente				
Sim	1,00	< 0,001	1,00	0,028
Não	1,38 (1,20–1,57)		1,20 (1,02–1,43)	

* Análise ajustada para sexo, cor da pele, classe econômica, escolaridade materna, região de origem, área do conhecimento, situação de moradia atual, local da última consulta odontológica, dor dentária e autopercepção da saúde bucal.

## DISCUSSÃO

A prevalência de uso regular de serviços odontológicos de 45,0% encontrada neste estudo de base universitária foi maior que a relatada em outros inquéritos de base populacional realizados no Brasil na mesma faixa etária. Não são do conhecimento dos autores estudos que tenham abordado a temática de uso regular de serviços odontológicos especificamente na população universitária brasileira. Em Pelotas, cidade em que está localizada a UFPel, um estudo de base populacional realizado por Camargo et al.^17^ em 2009 utilizou a mesma pergunta para mensurar o uso regular de serviços odontológicos e encontrou uma prevalência de 32,8% em adultos. Em um estudo mais recente, também de base populacional, realizado em Minas Gerais, a prevalência foi de 35,8% para o uso regular dos serviços odontológicos entre indivíduos maiores de 18 anos^13^. No estudo mineiro, indivíduos que tiveram a última consulta odontológica há mais de um ano foram classificados como uso irregular de serviços odontológicos. Entre aqueles que tiveram a última consulta odontológica há menos de um ano, o uso regular de serviços odontológicos foi definido com base em duas perguntas, uma relativa à periodicidade/regularidade das visitas e outra sobre o motivo da consulta. Indivíduos que consultaram o dentista no último ano e relataram uso de forma regular em ambas as perguntas subsequentes foram classificados como fazendo uso regular de serviços odontológicos.

É importante destacar que os estudos prévios utilizaram amostras de base populacional, enquanto a amostra de nosso estudo é composta por estudantes universitários. Apesar das políticas de inclusão universitária adotadas nos últimos anos, nosso estudo não reflete o perfil populacional brasileiro nessa faixa etária, pois não inclui populações em maior vulnerabilidade social, as quais têm os maiores problemas de saúde bucal e acessam menos os serviço de saúde^18^. Entretanto, no Brasil, houve uma expansão das políticas para a educação superior nas duas últimas décadas, com a inclusão dos grupos historicamente excluídos desse nível educacional, o que resultou na alteração significativa do perfil do estudante de graduação. Embora o processo de democratização da educação superior brasileira possa ser observado, continua tendo um sistema de acesso basicamente elitizado^3^. A prevalência mais elevada encontrada no presente estudo pode ser um reflexo dessa inclusão e mudança no perfil de universitários brasileiros, salientando uma política em prol da equidade social.

A associação entre classe econômica e uso regular de serviços odontológicos observada neste estudo concorda com a literatura atual, segundo a qual indivíduos mais pobres apresentam menor uso e maiores dificuldades no acesso aos serviços odontológicos^11^. Em um estudo realizado com 18 países selecionados da Organização para a Cooperação e Desenvolvimento Econômico (OCDE), todos eles apresentaram desigualdades significativas no acesso aos serviços odontológicos, relacionadas à renda^19^. Dessa forma, a classe econômica é um importante preditor no acesso à informação sobre hábitos e comportamentos preventivos em saúde^20^. Além disso, uma maior renda resulta na possibilidade de compra dos serviços de saúde^14^. Quando analisados os dados mundiais de gastos em saúde, os países com maior renda *per capita* são os que apresentam os maiores dispêndios em saúde bucal^21^. O mesmo processo que acontece entre os países ocorre entre os indivíduos^22^. Por essas razões, o uso regular dos serviços odontológicos entre os indivíduos mais vulneráveis economicamente se torna ainda mais difícil.

Fatores demográficos como sexo e cor da pele foram associados ao uso regular dos serviços odontológicos em diversos lugares do mundo, de acordo com a revisão sistemática e metanálise realizada por Reda et al.^11^. Contudo, nosso estudo encontrou associação estatística com a cor da pele na análise bruta apenas ao lidar com as perdas pela imputação múltipla dos dados faltantes, o que demostra que essa associação possivelmente existe, mas não tivemos poder para encontrá-la neste estudo. Já para o sexo, nem com a análise de imputação encontramos associação estatística, o que sugere que particularidades nas características da população universitária podem justificar a ausência de associação entre sexo e o uso regular de serviços odontológicos.

No que se refere às condições de saúde bucal autorreferidas, uma percepção positiva e a ausência de dor dentária estiveram associadas ao uso regular dos serviços odontológicos neste estudo. Corroborando esses achados, pode-se observar na literatura que a autopercepção positiva da saúde bucal^12,13,17^ e a ausência de dor de origem dentária^13^ foram associadas ao maior uso regular dos serviços odontológicos. Esses resultados podem ser explicados pelo fato de que a percepção de saúde pode ser considerada como um determinante para o entendimento da importância sobre as visitas regulares a um dentista e, consequentemente, mais ações preventivas resultam em menos episódios de dor e uma autopercepção positiva da saúde bucal.

O maior uso regular dos serviços odontológicos foi associado ao local da última consulta odontológica particular. No Brasil, Machado^12^ encontrou uma maior prevalência de uso regular naqueles que utilizaram serviços odontológicos privados de saúde que nos usuários de instituições públicas. No entanto, na Finlândia, após uma reforma nos serviços odontológicos públicos, foi observado um aumento do uso desses serviços nos últimos 12 meses, além de um crescimento de atendimentos na rede pública em relação à rede privada^23^.

Como limitações do presente estudo, podem ser apontadas as perdas e recusas diferenciais, maiores entre indivíduos do sexo masculino, mais velhos, matriculados em cursos da área de ciências exatas e da terra, ciências agrárias e engenharias e que viviam na região Sul do país anteriormente ao ingresso na UFPel, que poderiam resultar em viés de seleção. Devido ao desenho transversal do estudo, pode ocorrer viés de causalidade reversa para as associações entre o uso regular de serviços odontológicos e variáveis de saúde bucal, as quais podem ser bidirecionais. Outra possível limitação diz respeito à obtenção de informações autorreferidas sobre a regularidade das consultas odontológicas. Levando em consideração que se trata de um comportamento desejável socialmente, há possibilidade de superestimava do relato de consultas odontológicas por razões preventivas.

Como aspectos positivos deste estudo, destaca-se a forma da obtenção da regularidade do uso dos serviços odontológicos, uma vez que a maioria dos trabalhos que abordam a temática apenas investigam o tempo recordatório da última consulta odontológica para caracterizar a regularidade do hábito de frequentar o dentista, sem realizar distinção entre a motivação do indivíduo. Além disso, pode ser apontado como ponto forte a originalidade da população estudada.

Por fim, no presente estudo os estudantes relataram que utilizam mais o serviço para resolver problemas de saúde bucal e não de forma regular para prevenir agravos, como seria o desejável. Os resultados apontam para desigualdades no uso regular dos serviços odontológicos relacionadas a fatores socioeconômicos e um menor uso entre os universitários com piores condições de saúde bucal. Esses resultados sugerem que políticas públicas de prevenção e promoção em saúde em instituições de ensino superior devem ser realizadas para garantir qualidade de vida para esses jovens.
